# Use of vasopressin in the treatment of refractory septic shock

**DOI:** 10.5935/0103-507X.20180060

**Published:** 2018

**Authors:** Katiuce Tomazi Kny, Maria Angélica Pires Ferreira, Tatiane da Silva Dal Pizzol

**Affiliations:** 1 Núcleo de Avaliação e Tecnologias em Saúde, Hospital de Clínicas de Porto Alegre, Universidade Federal do Rio Grande do Sul - Porto Alegre (RS), Brasil.; 2 Departamento de Produção e Controle de Medicamentos, Faculdade de Farmácia, Universidade Federal do Rio Grande do Sul - Porto Alegre (RS), Brasil.

**Keywords:** Sepsis, Mortality, Hypotension, Vasopressin, Norepinephrine

## Abstract

**Objective:**

To evaluate the short-term evolution of patients with septic shock refractory to norepinephrine treated with vasopressin in an intensive care unit of a university hospital.

**Methods:**

An unmatched retrospective study (case series) was performed. Clinical, laboratory, and anthropometric data were collected from patients who received vasopressin infusion for treatment of catecholamine-refractory shock from December 2014 to June 2016. For the assessment of severity, APACHE II and SOFA scores were used. The main outcome was mortality at 3 and 30 days.

**Results:**

A total of 80 patients were included, of which 60% were male. In 86.3% of the cases, APACHE II was observed in the highest ranges (> 20). The 30-day mortality was 86.2%, and 75% of the patients died within 72 hours after starting vasopressin.

**Conclusion:**

The series evaluated had high mortality in the first 72 hours of treatment with vasopressin. The use of vasopressin in patients who are refractory to norepinephrine had little or no impact on mortality. It was not possible to exclude the possibility that the high mortality in the present study was linked to the relatively late onset (after established refractoriness of norepinephrine) of vasopressin; this hypothesis should be further evaluated in a randomized study.

## INTRODUCTION

Septic shock, defined by the need to use vasopressor to maintain mean blood pressure above 65mmHg after adequate infusion of fluids, associated with a serum lactate level above 2mmol/L, is the most common type of shock among hospitalized patients and an important cause of morbidity and mortality in Brazil and worldwide.^(1,2)^ In Brazil, the incidence of septic shock has increased in recent years; the 28-day mortality rate has reached approximately 50%, with an incidence density of 30 cases per thousand patients/day. According to the PROGRESS study, the overall lethality rate of septic shock is 49.6%; in Brazil, it is estimated that this rate reaches approximately 67%, with even higher rates in public hospitals.^(1-9)^

A prevalence study in 230 Brazilian intensive care units (ICUs) found that 30% of ICU beds in Brazil were occupied by patients with severe sepsis or septic shock.^(1,9)^ According to a report by the *Instituto Latino Americano da Sepse* (ILAS), 42.2% of patients hospitalized in public and private Brazilian hospitals in July 2015 died of complications and severity of sepsis.^(10,11)^ The mortality rate for septic shock with usual treatment (catecholamine use) varies in the range from 40 - 60%.^(12)^

Infusion of vasopressors in septic patients should be instituted whenever volume expansion is not sufficient to restore blood pressure and reverse organ dysfunction.^(6)^ According to international guidelines, the use of norepinephrine is recommended as the first choice vasopressor (recommended dose of 0.05 to 2µg/kg/min). A significant proportion of patients, however, do not achieve an adequate clinical response. Observational randomized clinical studies have shown that the administration of low doses of vasopressin in septic shock patients who are refractory to fluid replacement and the use of catecholamines may raise blood pressure and reduce the use of catecholamines; other potential physiological benefits are highlighted, such as a reduced risk of renal failure and arrhythmias.^(13-15)^

Thus, despite lacking high quality evidence showing a benefit in mortality, septic shock treatment guidelines recommend the addition of low dose vasopressin, corresponding to 0.03 - 0.04 International Units (IU)/minute, to norepinephrine as a therapeutic alternative in refractory cases, with the intention of increasing the mean arterial pressure (MAP) and decreasing the dose of norepinephrine.^(16)^ However, the effect of vasopressin on mortality remains controversial.^(17)^ More studies are needed to determine the best treatment strategy as well as which groups of patients would benefit most from the association of vasopressor with different mechanisms of action in this situation.

The present study aimed to evaluate the short-term evolution of patients with septic shock refractory to norepinephrine treated with vasopressin, in terms of mortality and length of stay in the ICU. The secondary objective was to describe the clinical characteristics of a series of cases with shock refractory to the first line of treatment.

## METHODS

An observational study of an unmatched retrospective design was performed. Data from patients who were hospitalized in the period from December 2014 to June 2016 were analyzed. Patients aged 18 years and older who were hospitalized in any hospital unit and started using vasopressin for the treatment of septic shock were included in the study. According to hospital policy, vasopressin in only released for the treatment of septic shock in cases that are refractory to norepinephrine, as defined by the attending physician. The patients were identified through a computerized prescription report, and those with a registry of dispensing and administration of vasopressin infusion were included. Data were collected on anthropometric measurements, baseline disease, duration of vasopressor use, presence of organ dysfunction, and complications. Patient data were collected directly from the electronic medical record, and evolution data were recorded until hospital outcome (discharge or death) or for up to 30 days after starting treatment with vasopressin.^(18)^

For the assessment of severity and likelihood of complications, the Acute Physiology and Chronic Health Evaluation II (APACHE II, obtained at the time of initiation of vasopressin therapy) and Sequential Organ Failure Assessment (SOFA) scores were recorded.^(19-21)^ To evaluate the correlation of mortality with SOFA, the Mann-Whitney test was used for independent samples.

The main outcomes were mortality at 3 and 30 days and length of ICU stay.

Data were collected using a standardized form, included in an Excel^®^ database, and analyzed quantitatively through the Statistical Package for Social Sciences (SPSS) software. The study was approved by the Research Ethics Committee of the *Hospital de Clínicas de Porto Alegre* (project: 150592; CAAE 51721915700005327).

## RESULTS

A total of 80 patients were included with a mean age of 55 years, and there was a predominance of the age group 61 years or older (42.5%), most of whom were men (60%). The clinical and demographic data of the sample are described in table 1. Most patients (86.3%) presented an APACHE score above 20 points; the median SOFA score obtained on the day of determined norepinephrine refractoriness was 11 points (25% percentile: 9; 75%: 13).

**Table 1:** Demographic and clinical characteristics of the analyzed patients (n = 80)

Variables	
Sex	
Male	60
Female	40
Age (years)	
< 25	3.8
26 - 40	17.5
41-60	36.3
≥ 61	42.5
APACHE II	
0 - 19	13.8
> 20	86.3
Infection sites	
Abdominal	37.5
Pulmonary	30
Not informed*	18.8
Renal/urinary	6.3
Heart	2.5
Pelvic	2.5
Multiple organs	1.3
Skin	1.3
Low heart rate	
Yes	53.7
No	46.3
Levels of SVO_2_	
Normal (68 - 77%)	2.5
Low	92.5
Not informed*	5
Mean blood pressure	
Normal	55
Hypotension (< 65mmHg)	45
Lactate levels	
Normal (1.0mmol/L to 1.8mmol/L)	22.5
High	73.8
Not informed	3.8
SOFA, p = 0.238	
Survivors up to 72 hours	10
Deaths up to 72 hours	11

APACHE - Acute Physiology and Chronic Health Evaluation; SVO_2_ - mixed venous saturation; SOFA - Sequential Organ Failure Assessment Score.

* Patients had no defined infection focus or it was not possible to identify these data in the medical record. Results expressed as % or median.

In all cases, the use of vasopressin followed the use of norepinephrine, which was used as the first option, in the usual dosage of 1µg/kg/minute.

The mean duration of norepinephrine treatment prior to initiation of vasopressin was 5 days. Vasopressin was used, on average, for 3 days, and the use was interrupted by death in most cases. At the time vasopressin was started, hemodialysis was performed in 26.3% of cases, and ventilatory failure was observed in 92.5% of cases.

Sixty patients (75%) died within 72 hours of initiation of vasopressin infusion. The 30-day mortality rate was 86.2% (Tables 2 and 3). There was no association between APACHE II score in relation to the incidence of death, with a lethality rate of 81.8% in the range of zero to 19 points and 78.3% in the range of >20 points. The same was observed using the SOFA score;^(22,23)^ a median of 11 (25% percentile: 9% and 75: 13) was observed in the group that died within 72 hours, and a median of 10 (25% percentile: 8 and 75%: 12.75) was observed in the group that survived (p = 0.238).

**Table 2:** Results (n = 80)

Results	
Days of use of norepinephrine (dose of 1µg/kg/min*)	
1 - 5	66.3
6 - 20	31.3
21 - 30	2.5
Days of use of vasopressin (dose of 0.03 - 0.04 IU/min*)	
1 - 5	95
6 - 20†	5
Days of ICU stay	
1 - 5	48.8
6 - 20	36.3
21 - 30	5.0
31 or more‡	10.0
Survival after vasopressin use 80 (%)	
1 - 5 days	59 (73.8)
6 - 20 days	10 (12.5)
21 - 30 days	1 (1.3)
31 days or more‡	10 (12.5)
Evolution in 3 days after use of vasopressin (in relation to response to treatment with vasopressin) 80 (%)	
Death	60 (75.0)
Improved	15 (18.8)
Worsened	4 (5.0)
Indifferent	1 (1.3)
Use of dobutamine	
Yes	6.3
No	93.8
Use of auxiliary therapy	
Total with at least one auxiliary therapy	98.8
Renal replacement	3.8
Invasive mechanical ventilation	3.8
Use of corticosteroids	5.0
Use of two auxiliary therapies	52.5
Use of three auxiliary therapies	33.7
Not informed	1.2
Period of introduction of vasopressin treatment	
After 48 hours of onset of sepsis	36.3
Ignored	1.3
Hospital outcome (within 30 days)	
Discharge	13.8
Death	86.2
Cause of death	
Related to sepsis	76.3
Other reasons	12.5

ICU - intensive care unit.

* Unit referring to the dosage of medicines;

† prescription authorized by the Drugs Commission of the Clinics Hospital of Porto Alegre;

‡ the length of hospital stay in the intensive care unit and the survival of patients over 30 days (maximum study period) cannot be stated. Results expressed as n (%).

**Table 3:** Clinical evolution of patients who survived after 72 hours (n = 19)

Evolution 30 days after vasopressin use	
Improved	47.4
Death	47.4
Worsened	5.2

Results expressed as %.

## DISCUSSION

Vasopressin was incorporated into the hospital's drug list in 2014 at the request of the Heart Transplant Service. Its use was approved for cases of shock associated with vasoplegia in the postoperative period of cardiac surgery. After inclusion on the list, however, vasopressin has also been prescribed in refractory cases of septic shock, especially in adults. In this way, the Pharmacy and Therapeutics Commission decided to evaluate the standard of use of the drug and its effectiveness when it was used outside the initially approved indication.

In the present study, a high mortality within 30 days was observed in patients who used vasopressin for the treatment of septic shock refractory to the use of norepinephrine. In fact, in several localized studies, mortality was lower than that observed in the present series. In the meta-analysis performed by Polito et al.,^(16)^ 40.6% mortality was verified in 512 septic patients who used vasopressin. However, that meta-analysis included studies in which vasopressin was used as a first-line therapy, while the present series evaluated its use in a situation of refractoriness to norepinephrine.

In a meta-analysis of randomized trials (32 studies, 3,544 patients) conducted by Avni et al.,^(24)^ the effect of different vasopressors on the total mortality of adult patients with septic shock was evaluated. With the exception of norepinephrine, which was associated with decreased mortality from all causes compared with dopamine (relative risk - RR 0.89; 95% confidence interval - 95%CI 0.81 - 0.98), no differences were observed in mortality among the different treatments. Hemodynamic outcomes were similar among the various vasopressors, with some superiority of norepinephrine in central venous pressure in urinary output and in lactate levels.

In a meta-analysis of randomized trials (9 studies, n = 998), Serpa Neto et al.^(25)^ observed a decrease in the need for norepinephrine among patients receiving vasopressin or terlipressin compared with controls (standard mean difference 1.58, 95%CI -1.73 - -1.44); p < 0.0001). In that study, the effect estimates are provided in combination for users of vasopressin and terlipressin.

Despite the limitations of APACHE II for predicting death, its use was approved given its widespread application in clinical practice as a parameter for severity and prognosis.^(19,26)^ The APACHE II score obtained after the analysis of cases showed that the highest incidence of death was in the range of > 20 points (69 patients), while the incidence of death in the range from zero to 19 points included 11 patients.

The follow-up of the individual evolution of patients through the medical records of the hospital occurred until the hospital outcome (discharge or death) or for a maximum period of 30 days after starting treatment with vasopressin.^(27,28)^ Therefore, for the patients who survived for a period of more than 30 days, the final outcome is not known, which may be considered as a limitation of the study. Among patients who survived more than 72 hours, only 18.8% showed improvement. This finding provide an explanation for why randomized clinical trials usually have a short follow-up time, as in the case of the studies by Malay et al.^(14)^ and Patel et al.,^(15)^ with follow-up times of 4 and 24 hours, respectively. However, there are other cases with a prolonged follow-up, such as in the study by Russell et al.^(17)^ in which mortality was assessed at 28 and 90 days.

Another limitation is that during the study, detailed measurements of important hemodynamic and metabolic outcomes, such as blood levels of lactate, cytokines, and troponins, were not performed; thus, there is the need for further studies to evaluate these variables. It was chosen not to evaluate these parameters in detail but instead to evaluate a "difficult" outcome (mortality). The clinical response to the vasopressor effect was evaluated through MAP.^(15)^

Given the uncontrolled design, this study can be considered to be predominantly exploratory and hypothesis-generating in nature; in fact, based on these data, it is not possible to establish the efficacy of vasopressin for reducing mortality. The study is, however, considered useful for reflections on clinical practice, given the frequent use of vasopressin in this context, despite the lack of good quality evidence in situations of refractoriness.

## CONCLUSION

Early mortality was elevated in septic patients with refractory shock treated with vasopressin. The high rate of therapeutic failure may have been due to the severity profile of the baseline disease; another possibility could be the relatively late introduction of vasopressin. However, the association of vasopressin with first-line catecholamines has not been shown to be effective in clinical studies.
